# Prevalence of Diabetic Peripheral Neuropathy and Its Associated Risk Factors Among Patients With Type 2 Diabetes Mellitus in the Chengalpattu District of Tamil Nadu

**DOI:** 10.7759/cureus.83586

**Published:** 2025-05-06

**Authors:** Gowtham S, Anantha Eashwar VM, Ilamilaval Thozhanenjan, Nikhil CM, Kiruthika Narayanan

**Affiliations:** 1 Community Medicine, Sree Balaji Medical College and Hospital, Chennai, IND

**Keywords:** complications, disability, glycated hemoglobin (hba1c), hypertension, medicine adherence

## Abstract

Background

Diabetic peripheral neuropathy (DPN) is a common diabetic consequence that damages nerves and results in pain, numbness, and sensory loss. It has no known treatment, impairs mobility, and increases the risk of foot ulcers; therefore, early therapy is necessary. The objective of this study was to determine the factors associated with DPN in individuals with type 2 diabetes mellitus (T2DM).

Methodology

A cross-sectional study was conducted from July 2024 to December 2024 among 310 people who attended the diabetic outpatient department (OPD) of a tertiary care hospital in Chengalpattu district. The Neuropathy Disability Score (NDS), Neuropathy Symptom Score (NSS), and Morisky Medication Adherence Scale-8 (MMAS-8) were used, and data were collected and analyzed using IBM SPSS Statistics for Windows, V. 25.0 (IBM Corp., Armonk, NY, USA). Analytical tests such as the chi-squared test and odds ratio were used to find an association between DPN and its associated variables such as age, sex, glycated hemoglobin (HbA1c), and the duration of illness following which the enter method of logistic regression analysis was performed.

Results

Out of 310 participants, the prevalence of DPN was found to be present in 88 (28.4%). DPN was found to be significantly associated with education (adjusted odds ratio (AOR): 0.33, 95%CI: 0.15-0.74), marital status (AOR: 2.88, 95%CI: 1.52-5.47), comorbidities (AOR: 2.07, 95%CI: 1.05-4.08), and type of medication (AOR: 2.59, 95%CI: 1.29-5.21).

Conclusion

About 88 (28.4%) of people had DPN; those who were hypertensive and used insulin to control their DM were at a greater risk. Routine screening and control of blood sugar and blood pressure are essential for patients with DM.

## Introduction

The American Diabetes Association defines diabetes mellitus (DM) as "a group of metabolic diseases characterized by hyperglycemia resulting from defects in insulin secretion, insulin action, or both" [1]. According to the International Diabetes Federation Diabetes Atlas, 10.5% of the global adult population aged 20-79 years has DM, with nearly half unaware of their condition. Projections indicate that by 2045, approximately 783 million adults, or one in eight individuals, will be living with DM, representing a 46% increase. More than 90% of these cases are of type 2 DM (T2DM), influenced by socioeconomic, demographic, environmental, and genetic factors [2]. In a meta-analysis done in China, 29 studies with a total of 50,112 participants were included. The results showed that the pooled prevalence of diabetic peripheral neuropathy (DPN) was 30% [3]. Another study reported a prevalence of DPN between 6% and 51% depending on age, increasing DM duration, and the level of glycemic control [4]. The prevalence of DPN in India varies significantly, with estimates ranging from 9.6% to 78% across populations. Studies have reported a prevalence of 29.2% and 47% in Northern and Southern India, respectively. This wide variation underscores the need for region-specific studies and tailored interventions to address unique challenges faced by different populations [5].

One of the most common complications of DM is peripheral neuropathy, which refers to a spectrum of clinical conditions that involve peripheral nervous system dysfunction. Patients commonly experience symptoms such as numbness, tingling, burning sensations, muscle weakness, hyperalgesia, allodynia, and pain. DPN, the most common subtype, can result in severe complications including paresthesia, limb loss, and even mortality. Peripheral and autonomic neuropathies are some of the leading causes of morbidity in DM. Studies have shown that at five years, the risk of death for patients with a diabetic foot ulcer is 2.5 times as that for patients with DM who do not have a foot ulcer. This stark statistic highlights the severe impact of DPN and its related complications on patient outcomes and emphasizes the importance of early diagnosis and management [6].

The major problem with the development of DPN is that people often ignore the signs of nerve damage because they believe that it is a part of aging [7]. Available evidence shows that individuals with DPN experience a decreased quality of life [8]. Screening of DPN in the clinical practice using a simple objective tool is essential, as the detection of various soft and subtle signs of DPN at the earliest could minimalize the damaging effects of serious complications [9]. 

In conclusion, understanding the prevalence of DPN among individuals with T2DM in India is crucial because of the increasing burden of DM and its associated complications. Identifying the associated factors can aid in the development of targeted interventions and preventive measures, ultimately improving the quality of life of individuals affected by this condition. Therefore, this study aimed to estimate the prevalence of DPN and its risk factors among patients with T2DM.

## Materials and methods

Study design

The present study was a cross-sectional study conducted between the months of July 2024 and December 2024 among T2DM patients who are 18 years and above and attending a diabetic outpatient department (OPD) in Sree Balaji Medical College and Hospital, Chennai, India. Bedridden patients and patients with peripheral nerve injury, leprosy, stroke, and severe communication impairments were excluded.

Sample size

Based on a study done by Darivemula et al., the prevalence of DPN was found to be 39.3% [10]. Considering this as prevalence (p) and applying the formula n=Z^2^pq/d^2^, with Z=1.96, p=39.3%, q=(100-p)=60.7%, and allowable error (d)=6%, the sample size was 254.5. Allowing a 20% non-responsive rate, the required sample size is 305, and the final sample size was rounded to 310.

Sampling method

Simple random sampling was used. A list of eligible T2DM patients attending the diabetic OPD in a tertiary care hospital with recent blood reports during the study period was compiled. From this list, participants were selected using a random number generator to ensure every patient had an equal chance of being included in the study.

Ethical approval

Approval was granted by the Institutional Human Ethics Committee of Sree Balaji Medical College and Hospital under reference number 002/SBMCH/IHEC/2024/2243.

Data collection tools

To collect demographic information, a pretested structured questionnaire was used. Validated questionnaires like the Neuropathy Disability Score (NDS) and the Neuropathy Symptom Score (NSS) were used to assess DPN, while the Morisky Medication Adherence Scale-8 (MMAS-8) was used to assess the medicine adherence of the participants. No trained staff or assistants were used for data collection.

NDS

The NDS was used for the neurological assessment, including four clinical tests on both feet. Before the assessment, the patient's hand was examined and the procedure was explained. The patient closed their eyes during the examination. To determine the overall impairment score, each test was assessed using a point scale. The following clinical examinations were carried out: A 10 g monofilament was applied on various sites of the sole to assess pressure feeling. The ability to feel pressure might have also been evaluated in other bone prominences, including the metatarsal heads. By placing a 128 Hz tuning fork on many bony prominences, including the big toe's hallux, vibration perception was examined. Applying a pinprick with sufficient force to slightly depress the skin in many places, including the proximal region of the big toe, allowed researchers to measure how much pain was felt. To assess pressure feeling in a foot with hypoesthesia, the monofilament test was used. "Absent" results indicate that the patient did not perceive the pinprick's severity, whereas "present" results indicate that the participant felt its intensity. If the three emotions were present and normal, they were scored 0; if they were absent, decreased, or unknown, they were scored 1. Using a conventional patellar hammer technique, the Achilles deep tendon reflex was examined. A score of 0 meant normal function; a score of 1+ meant reinforcement; and a score of 2+ meant absence. The score of the examined foot would be doubled if it had previously been severed. To determine the severity of diabetic nephropathy (DN), the NDS provides a score range of 0-10. Three levels of severity were established for neuropathy handicap: mild (3-5), moderate (6-8), and severe (9-10) [11].

NSS

The NSS is a commonly used scale to determine neurological symptoms such as burning sensation, numbness, and tingling, as well as symptoms of fatigue, cramps, and aches. The NSS score is from 0 to 9, where 0-2 means normal, 3-4 mild, 5-6 moderate, and 7-9 severe [12].

MMAS-8

Using the MMAS-8, medication adherence was evaluated. The scale has eight questions, the first seven of which have a yes/no response that indicates whether the respondent is adhering to the rules or not. On item 8, patients can select a response on a 5-point Likert scale that indicates how frequently they forget to take their prescriptions. Scores on the MMAS-8 might be anywhere between 0 and 8. Individuals with adherence scores of 8 points, <8 to >6 points, and <6 points were categorized as having high, medium, and low adherence, respectively [13].

Data analysis

Data was analyzed using IBM SPSS Statistics for Windows, V. 25.0 (IBM Corp., Armonk, NY, USA). Statistical analysis included the chi-squared test to find an association, and logistic regression was done to find the odds ratio (OR).

## Results

Out of 310 study participants, 136 (43.9%) were less than 60 years of age. More than half of the participants were female (159, 51.3%), and the majority had glycated hemoglobin (HbA1c) >7 (236, 76.1%). Almost 99 (31.9%) respondents were graduates, and the majority of the participants were unemployed (207, 66.8%). Approximately 205 (66.1%) patients had a family history of DM (Table 1).

**Table 1:** Sociodemographic characteristics of participants (n=310) Data are presented as n (%), where n represents the number of responses in each variable and (%) indicates the percentage of participants, respectively. HbA1c: glycated hemoglobin; BMI: body mass index; T2DM: type 2 diabetes mellitus; OHA: oral hypoglycemic agents

Variable	Female n (%)	Male n (%)	Participants n (%)
Age (years)			
<60	64 (47.1)	72 (25.9)	136 (43.9)
>60	95 (54.6)	79 (45.4)	174 (56.1)
Gender	159 (51.3)	151 (48.7)	310 (100)
Religion			
Hindu	91 (50.3)	90 (49.7)	181 (58.4)
Christian	42 (57.5)	31 (42.5)	73 (23.5)
Muslim	26 (46.4)	30 (53.6)	56 (18.1)
Residence			
Urban	96 (51.6)	90 (48.4)	186 (60)
Rural	63 (50.8)	61 (49.2)	124 (40)
Education			
Illiterate	66 (60)	44 (40)	110 (35.5)
School education	49 (48.5)	52 (51.5)	101 (32.6)
Graduate	44 (44.4)	55 (55.6)	99 (31.9)
Occupation			
Unemployed	77 (37.2)	130 (62.8)	207 (66.8)
Job/business	82 (79.6)	21 (20.4)	103 (33.2)
Marital status			
Married	26 (53.1)	23 (46.9)	49 (15.8)
Single	121 (53.5)	105 (46.5)	226 (72.9)
Widow/divorced	12 (34.3)	23 (65.7)	35 (11.3)
Living status			
Alone	35 (53)	31 (47)	66 (21.3)
With children	17 (51.5)	16 (48.5)	33 (10.6)
With family	99 (55.9)	78 (44.1)	177 (57.1)
With spouse	8 (23.5)	26 (76.5)	34 (11)
Financial dependence			
Dependent	87 (68.5)	40 (31.5)	127 (41)
Independent	72 (39.3)	111 (60.7)	183 (59)
HbA1c			
>7	120 (50.8)	116 (49.2)	236 (76.1)
<7	39 (52.7)	32 (47.3)	74 (23.9)
BMI			
Obesity	21 (58.3)	15 (41.7)	36 (11.6)
Overweight	45 (52.3)	41 (47.7)	86 (27.7)
Underweight	18 (52.9)	16 (47.1)	34 (11)
Normal	75 (48.7)	79 (51.3)	154 (49.7)
Comorbidities			
Hypertension	44 (54.3)	37 (45.7)	81 (26.1)
Increased cholesterol	19 (51.4)	18 (48.6)	37 (11.9)
Both	15 (37.5)	25 (62.5)	40 (12.9)
None	81 (53.3)	71 (46.7)	152 (49)
Smoking			
Yes	27 (26.7)	74 (73.3)	101 (32.6)
No	132 (63.2)	77 (36.8)	209 (67.4)
Alcohol			
Yes	26 (31.3)	57 (68.7)	83 (26.8)
No	133 (58.6)	94 (41.4)	227 (73.2)
Family history of T2DM			
Yes	104 (50.7)	101 (49.3)	205 (66.1)
No	55 (52.4)	50 (47.6)	105 (33.9)
Duration of illness			
>10 years	98 (52.4)	89 (47.6)	187 (60.3)
<10 years	61 (49.6)	62 (50.4)	123 (39.7)
Type of medication			
OHA and insulin	28 (21.9)	26 (48.1)	54 (17.4)
Insulin	32 (50.8)	31 (49.2)	63 (20.3)
OHA	99 (51.3)	94 (48.7)	193 (62.3)

Table 2 shows the association of DPN with sociodemographic variables. On bivariate analysis, the factors found to be statistically significant were rural (OR: 1.77, 95%CI: 1.07-2.92), school education (OR: 1.89, 95%CI: 1.04-3.43), marital status (OR: 2.88, 95%CI: 1.52-5.47), HbA1c >7 (OR: 2.18, 95%CI: 1.13-4.22), hypertension (OR: 2.35, 95%CI: 1.30-4.24), smoking (OR: 2.35, 95%CI: 1.41-3.90), family history of DM (OR: 1.82, 95%CI: 1.08-3.06), duration of DM (OR: 1.88, 95%CI: 1.10-3.18), and type of medication (OR: 1.91, 95%CI: 1.04-3.50).

**Table 2:** Association of sociodemographic variables with diabetic peripheral neuropathy Data are presented as n (%), where n represents responses and (%) indicates the percentage of participants. The chi-squared test and odds ratio were used to test the association at 95%CI. *p<0.05: statistically significant. 95%CI: 95% confidence interval; HbA1c: glycated hemoglobin; BMI: body mass index; T2DM: type 2 diabetes mellitus; OHA: oral hypoglycemic agents

Variable	Diabetic peripheral neuropathy (n=310)		Odds ratio	95%CI	Chi-squared test	P-value^*^
	Present n (%)	Absent n (%)				
Age (years)						
>60	43 (31.6)	93 (68.4)	1.32	0.80-2.17	1.24	0.26
<60	45 (25.9)	129 (74.1)	-	-	-	-
Gender						
Female	50 (31.4)	109 (68.6)	1.36	0.82-2.24	1.50	0.22
Male	38 (25.2)	113 (74.8)	-	-	-	-
Religion						
Hindu	46 (25.4)	135 (74.6)	0.85	0.43-1.66	0.22	0.63
Christian	26 (35.6)	47 (64.4)	1.38	0.65-2.93	0.71	0.39
Muslim	16 (28.6)	40 (71.4)	-	-	-	-
Residence						
Urban	44 (23.7)	80 (64.5)	-	-	-	-
Rural	44 (35.5)	142 (76.3)	1.77	1.07-2.92	5.12	0.024^*^
Education						
Illiterate	19 (17.3)	91 (82.7)	0.55	0.28-1.08	3.03	0.08
School education	42 (41.6)	59 (58.4)	1.89	1.04-3.43	4.53	0.034^*^
Graduate	27 (27.3)	72 (72.7)	-	-	-	-
Occupation						
Unemployed	60 (29)	147 (71)	1.09	0.64-1.85	0.1	0.74
Job/business	28 (27.2)	75 (72.8)	-	-	-	-
Marital status						
Married	23 (46.9)	26 (53.1)	2.88	1.52-5.47	11.1	0.001^*^
Widow/divorced	12 (34.3)	23 (65.7)	1.7	0.79-3.65	1.9	0.17
Single	53 (23.5)	173 (76.5)	-	-	-	-
Living status						
Alone	27 (40.9)	39 (59.1)	1.44	0.60-3.45	0.69	0.40
With children	6 (18.2)	27 (81.8)	0.46	0.14-1.45	1.77	0.18
With family	44 (24.9)	133 (75.1)	0.69	0.31-1.53	0.83	0.36
With spouse	11 (32.4)	23 (67.6)	-	-	-	-
Financial dependence						
Dependent	35 (27.6)	92 (72.4)	0.93	0.56-1.54	0.07	0.78
Independent	53 (29)	130 (71)	-	-	-	-
HbA1c						
>7	75 (31.8)	161 (68.2)	2.18	1.13-4.22	5.59	0.01^*^
<7	13 (17.6)	61 (82.4)	-	-	-	-
BMI						
Obesity	11 (30.6)	25 (69.4)	1.06	0.48-2.34	0.02	0.87
Overweight	22 (25.6)	64 (74.4)	0.83	0.45-1.51	0.36	0.54
Underweight	10 (29.4)	24 (70.6)	1.00	0.44-2.28	0.00	0.98
Normal	45 (29.2)	109 (70.8)	-	-	-	-
Comorbidities						
Hypertension	32 (39.5)	49 (60.5)	2.35	1.30-4.24	8.31	0.004^*^
Increased cholesterol	11 (29.7)	26 (70.3)	1.52	0.68-3.40	1.07	0.30
Both	12 (30)	28 (70)	1.54	0.70-3.36	1.21	0.27
None	33 (21.7)	119 (78.3)	-	-	-	-
Smoking						
Yes	41 (40.6)	60 (59.4)	2.35	1.41-3.90	10.98	0.001^*^
No	47 (22.5)	162 (77.5)	-	-	-	-
Alcohol						
Yes	24 (28.9)	59 (71.1)	1.03	0.59-1.80	0.01	0.90
No	64 (28.2)	163 (71.8)	-	-		-
Family history of T2DM						
Yes	60 (24.4)	120 (75.6)	1.82	1.08-3.06	5.16	0.02^*^
No	28 (36.2)	102 (63.8)	-	-	-	-
Duration						
>10 years	62 (33.2)	125 (66.8)	1.88	1.10-3.18	5.27	0.02^*^
<10 years	26 (21.1)	97 (78.9)	-	-	-	-
Type of medication						
OHA and insulin	17 (31.5)	37 (68.5)	1.42	0.73-2.76	1.11	0.292
Insulin	24 (38.1)	39 (61.9)	1.91	1.04-3.50	4.47	0.03^*^
OHA	47 (24.4)	146 (75.6)	-	-	-	-

Table 3 shows a significant association between DPN and MMAS-8 (OR: 3.18, 95%CI: 1.63-6.20).

**Table 3:** Association of diabetic peripheral neuropathy with MMAS-8 Data are presented as n (%), where n represents responses and (%) indicates the percentage of participants. The chi-squared test and odds ratio were used to test the association at 95%CI. *p<0.05: statistically significant. 95%CI: 95% confidence interval; MMAS-8: Morisky Medication Adherence Scale-8

Variable	Diabetic peripheral neuropathy		Odds ratio	95%CI	Chi-squared test	P-value
	Present (%)	Absent (%)				
MMAS-8 category						
High adherence	25 (41.7)	35 (58.3)	-	-	-	-
Medium adherence	37 (34.3)	71 (65.7)	1.37	0.71-2.62	0.90	0.95
Low adherence	26 (18.3)	116 (81.7)	3.18	1.63-6.20	12.19	0.00^*^

Table 4 shows the results of logistic regression analysis. On logistic regression analysis, the following factors were found to have a statistically significant association (p<0.05): education (adjusted odds ratio (AOR): 0.33, 95%CI: 0.15-0.74), marital status (AOR: 3.27, 95%CI: 1.52-7.03), hypertension (AOR: 2.07, 95%CI: 1.05-4.08), and insulin therapy (AOR: 2.59, 95%CI: 1.29-5.21).

**Table 4:** Logistic regression analysis of diabetic peripheral neuropathy and related variables Data are presented as n (%), where n represents responses and (%) indicates the percentage of participants. Logistic regression was used to test the association at 95%CI. **p<0.05: statistically significant 95%CI: 95% confidence interval; AOR: adjusted odds ratio; HbA1c: glycated hemoglobin; OHA: oral hypoglycemic agents

Variable	AOR	95%CI	P-value^**^
Residence			
Rural	-	-	-
Urban	0.73	0.40-1.33	0.31
Education			
Graduate	-	-	-
School education	1.30	0.66-2.56	0.43
Illiterate	0.33	0.15-0.74	0.00^**^
Marital status			
Single	-	-	-
Separated/divorced	1.11	0.47-2.65	0.80
Married	3.27	1.52-7.03	0.00^**^
HbA1c			
<7	-	-	-
>7	1.61	0.79-3.31	0.18
Comorbidities			
None	-	-	-
Both	1.36	0.56-3.30	0.49
Increased cholesterol	1.45	0.58-3.61	0.41
Hypertension	2.07	1.05-4.08	0.03^**^
Smoking			
No	-	-	-
Yes	0.58	0.30-1.12	0.10
Family history			
No	-	-	-
Yes	0.95	0.52-1.73	0.87
Duration of illness			
<10 years	-	-	-
>10 years	1.63	0.86-3.11	0.13
Type of medication			
OHA	-	-	-
Insulin	2.59	1.29-5.21	0.00^**^
Both	1.09	0.48-2.46	0.82

## Discussion

The primary objective of this study was to determine the prevalence of DPN. From this perspective, the prevalence of DPN was 88 (28.4%) in our study. In a study conducted by Bhuyan and Appaiah in Assam, the prevalence of DPN in newly diagnosed T2DM patients in India was 55 (68.75%) [14]. In a study conducted by Solanki et al., the prevalence among urban type 2 diabetics in Gujarat, India, was 38 (38%). This highlights the significant impact of DPN as a common microvascular complication in the Indian diabetic population [15]. In a study conducted by Chander in Jammu, the prevalence of newly diagnosed T2DM patients in rural Northern India was 29 (29%) [16]. In a study conducted by Bansal et al. in Chandigarh, the prevalence was 171 (29.2%) [17]. In a study conducted by Perveen et al., the prevalence of peripheral neuropathy among patients with DM was 100 (44.4%) [18]. The variance in the prevalence of DPN may be due to varying study locations and the use of different methods in measuring DPN. 

This study showed a statistically significant association between DPN and HbA1c. Likewise, in a study conducted by Deekshanya et al. in Puducherry, there was a significant association between HbA1c levels and the prevalence of DPN in asymptomatic patients with T2DM [19]. A study done by Zeno et al. in India found a statistical significance between high HbA1c values and the presence of subclinical peripheral neuropathy in DM patients, indicating that elevated HbA1c levels are associated with a higher prevalence of DPN in India [20]. In a study conducted by Cheng et al. in China, there was a significant association between HbA1c levels and the prevalence of DPN, with HbA1c >7.7% increasing the risk of DPN in adult patients with T2DM [21]. In a study conducted by Bhuyan and Appaiah in Assam, HbA1c showed a significant association with the prevalence of DPN in newly diagnosed T2DM patients with higher levels correlating with increased neuropathy severity [14]. These results show that individuals with poorly controlled HbA1c have a high risk of developing DPN.

In the present study, there was a significant association between hypertension, smoking, and the prevalence of DPN. In a study conducted by Cheng et al., a history of hypertension was statistically significant [21]. In a study conducted by Abdulla Ebrahim et al. in Egypt, significant comorbidities associated with DPN included dyslipidemia, abdominal obesity, and longer duration of DM [22]. In a study conducted by Dinh Le et al. in Vietnam, comorbidities associated with DPN included age, smoking, hypertension, poor HbA1c control, triglycerides, albumin levels, 24-hour urinary albumin, and diabetic retinopathy [23]. In a study conducted by Khawaja et al., comorbidities significantly associated with DPN include unemployment, cardiovascular disease, and dyslipidemia [24]. In a study conducted by Aleidan et al., in Saudi Arabia, comorbidities significantly associated with DPN included hypertension, dyslipidemia, and increased body mass index (BMI) [25]. In a study conducted by Meena and Manikandan in Tamil Nadu, comorbidities associated with higher prevalence included age over 50, female sex, DM duration exceeding 10 years, HbA1c levels above 6.5, and BMI over 23 [26]. In a study conducted by Abdissa et al. in Ethiopia, comorbidities associated with DPN included advanced age, prolonged DM duration, physical inactivity, and smoking, highlighting the need for early detection and intervention [27]. However, we did not find any association with BMI, dyslipidemia, age, unemployment, and cardiovascular diseases. These differences may be due to the individual's lifestyle patterns followed in different regions.

In this study, low medication adherence was associated with the prevalence of DPN. In a study conducted by Chakraborty and Majumder in West Bengal, several correlates associated with DPN were identified, including poor medication adherence, which was assessed using a medication adherence rating scale [28]. In a study conducted by Zhang et al., 268 (59.8%) had medication non-adherence, and 97 (21.7%) were non-adherent to scheduled appointments. The Problem Areas in Diabetes (PAID) score, peripheral neuropathy, home glucose monitoring, HbA1c, and age were associated with medication non-adherence [29]. In a study done by Mirahmadizadeh et al., associated factors such as 50-64 years of age, at least 65 years of age, overweight, obesity, divorced widows, smokers, and ex-smokers had a significant influence on adherence to medication [30]. From these studies, it is evident that low medicine adherence in diabetics leads to the development of DPN. Further studies should be conducted to find the factors that lead to low medicine adherence.

This study showed a significant association between insulin medication and DPN. A study conducted by El-Bably et al. in Egypt found that insulin therapy was significantly associated with DPN, indicating that insulin use increases the risk of developing this complication in diabetic patients [31]. A study done by Young et al. in Nigeria found that painful DPN prevalence was 121 (44.5%) among diabetic patients, with a significant association with insulin therapy [32]. Similarities between the studies are because the individuals in insulin therapy tend to have uncontrolled DM which leads to higher chances of getting DPN.

Limitations

This is a facility-based cross-sectional study focused on patients with DM attending hospitals for follow-up and care. As a result, no cause-and-effect relationship can be established, and the results cannot be generalized. Hospital-based study setting may induce selection bias, limiting the application of the results to a wider population.

## Conclusions

The study's findings revealed that residence, education, marital status, HbA1c, comorbidities, smoking, family history of DM, duration of DM, and type of medication were all significantly associated with an increased risk of DPN among diabetic patients. Using machine learning models, the risk of DPN can be analyzed with biochemical data. Also, to enhance medicine adherence, digital health tools can be developed. Regular screening for blood sugar levels and blood pressure should be performed in patients with DM. Individuals undergoing insulin therapy should be regularly monitored. Health education regarding diet, physical activity, and foot care can be provided.
